# The lag-phase during diauxic growth is a trade-off between fast adaptation and high growth rate

**DOI:** 10.1038/srep25191

**Published:** 2016-04-29

**Authors:** Dominique Chu, David J. Barnes

**Affiliations:** 1 University of Kent, School of Computing, Canterbury, CT2 7NF, UK

## Abstract

Bi-phasic or diauxic growth is often observed when microbes are grown in a chemically defined medium containing two sugars (for example glucose and lactose). Typically, the two growth stages are separated by an often lengthy phase of arrested growth, the so-called lag-phase. Diauxic growth is usually interpreted as an adaptation to maximise population growth in multi-nutrient environments. However, the lag-phase implies a substantial loss of growth during the switch-over. It therefore remains unexplained why the lag-phase is adaptive. Here we show by means of a stochastic simulation model based on the bacterial PTS system that it is not possible to shorten the lag-phase without incurring a permanent growth-penalty. Mechanistically, this is due to the inherent and well established limitations of biological sensors to operate efficiently at a given resource cost. Hence, there is a trade-off between lost growth during the diauxic switch and the long-term growth potential of the cell. Using simulated evolution we predict that the lag-phase will evolve depending on the distribution of conditions experienced during adaptation. In environments where switching is less frequently required, the lag-phase will evolve to be longer whereas, in frequently changing environments, the lag-phase will evolve to be shorter.

Diauxic growth is the phenomenon whereby a population of microbes, when presented with two carbon sources, exhibits bi-phasic exponential growth intermitted by a *lag-phase* of minimal growth. Originally, the phenomenon was described by Monod^1^ demonstrating diauxie with glucose and lactose in *E. coli* . In his experiments Monod showed that the population first grows exponentially on glucose until all glucose is exhausted, then stops growing for a considerable amount of time and subsequently resumes exponential growth on lactose. The duration of the lag-phase can be substantial (order of magnitude of a generation time).

Diauxic growth and the network that controls it has since been subject to intense experimental^2^,^3^,^4^,^5^,^6^,^7^,^8^ and theoretical^5^,^9^,^10^,^11^ investigation. There are two main mechanisms responsible for two phase growth in bacteria: (*i*) Regulation of metabolic genes via global transcription regulators, especially cAMP. (*ii*) Direct repression of uptake of the secondary nutrient in the presence of glucose.

Both of those effects are mediated by the phosphotransferase system (PTS)^12^, whereby a phosphoryl group (P) is transferred from PEP to the carbohydrate in a five step phosphorelay. In Enterobacteriaceae the PTS relay is catalysed by the generic “Enzyme I” (EI) and HPr as well as the carbohydrate specific components EIIA, EIIB, EIIC. During glucose uptake the levels of dephospho EIIA^Glc^ increase. In its dephosphorylated form this enzyme directly interacts with the permeases of the secondary sugars (i.e. lactose). In this way it prevents nutrient uptake and thus the induction of the relevant uptake system (inducer exclusion).

Diauxic growth has traditionally been interpreted as an adaptation that allows the maximisation of growth in dual-nutrient environments. Intuitively, the reasoning is like so: When there are two shared resources, one good, one less good, then sequential uptake of nutrient can be shown to be a beneficial strategy under a wide range of conditions^13^,^14^. What remains unclear is why the lag-phase evolved. Halting exponential growth even for short periods of time entails a significant loss of fitness (i.e. number of offspring produced) relative to competitors that do not halt their growth. The fact that there is a lag-phase therefore requires an explanation.

One hypothesis is that the lag-phase is a direct consequence of the time required to switch on the relevant metabolic genes of the system^15^,^16^ and that it therefore is an unavoidable “engineering” constraint of the system. It is unclear how relevant this hypothesis is because inherent delays in gene expression can always be compensated by inducing the genes *before* the primary nutrient is exhausted, thus avoiding any period of arrested growth altogether. Hence, the hypothesis that the origin of the lag-phase is simply a consequence of the time required to activate genes expression does not provide much insight. It is also contradicted by recent experimental evidence showing (*i*) that the time required to induce genes does not seem to determine the length of the lag-phase^17^,^18^ and (*ii*) that the lag-phase is under evolutionary control^8^,^19^. This latter observation points to an adaptive role of the lag-phase, in the sense that the cell gains some benefit from it. *Prima facia* it is unclear in what sense a prolonged period of arrested growth can contribute to fitness in micro-organisms.

Recent observations of single-cell dynamics during the switch from one nutrient to another provide some hints towards a better understanding of the evolution of the lag-phase. For both yeast^20^ and *E. coli*^4^ it was shown that the population is heterogeneous with respect to the activation state of the primary and secondary metabolism. During the lag-phase there are cells that grow on both the primary and the secondary nutrient. It appears that the lag-phase is the result of an unequal distribution of growth rates within the population^4^,^18^,^21^ rather than a reflection of the typical cell behaviour.

Crucial for the understanding of the evolutionary significance of the lag-phase is also a recent observation by Wang *et al.*^17^. They found that switching to the secondary carbon source starts even before the primary nutrient is exhausted—during the so-called *preparation phase* and that this preparation phase determines the duration of the lag-phase. The authors also established that the growth rate on the primary nutrient and the duration of the preparation phase are correlated. This finding is intriguing because it relates the maximal growth rate of the cells to the duration of the lag-phase, which suggests that the lag-phase is a manifestation of an evolutionary trade-off between the growth rate and the ability to adapt to new conditions. Similar trade-offs seem to be a general feature of biological systems appearing widely across many scales, including metabolic fluxes^18^,^22^, gene regulation^23^ or sensory systems^24^. While the trade-off between adapting fast and growing fast is well supported by experimental evidence, so far the mechanistic origin of the trade-off remains unclear.

In this contribution we will show that the evolutionary significance of the lag-phase can be understood when the diauxic shift is cast as a sensing problem. This perspective becomes natural once one recognises that the cell is only able to shift from the primary nutrient to the secondary nutrient if it has sensed that the primary nutrient is exhausted and that a secondary nutrient is present. This view is interesting because it opens up for the possibility to explain the lag-phase as a consequence of fundamental (and well known) limitations of biological sensors: The ability to sense external nutrient concentrations with a given degree of accuracy requires a corresponding minimal energy expenditure^25^,^26^. This is relevant to diauxic growth because the switching speed of individual cells is limited by the ability of the cell to detect (*i*) the concentration of the primary nutrient as it is depleting and (*ii*) the presence of a particular secondary nutrient. The cost of this sensing caps permanently the ability of the cell to grow on any carbon source.

The hypothesis proposed here is that poor quality sensing, while cheap, entails inefficient switching between the two nutrient sources and thus a lag-phase. More accurate sensing is more expensive and reduces growth at all times, but enables the cells to switch faster to a new nutrient source. Based on this, one would then expect that there is an optimal lag-phase, that depends on the characteristics of the environment. In environments that rarely change, it should be better to grow rapidly but switch slowly. *Vice versa* in environments that see a frequent change of conditions, it would be beneficial to be able to adapt rapidly.

We support our hypothesis by means of extensive simulations using a minimal stochastic computer model based on the bacterial PTS system. While the model assumes generic de-repression motifs of metabolic systems as they frequently occur in bacteria, it also abstracts from the details of microbial synthesis pathways. The resulting minimal model allows us to make the connection between the lag-phase and well-known, general, limitations of biological sensors.

Using extensive parameter sweeps, we show that this model predicts empirically known correlations between growth-rate and the length of the lag-phase, and population heterogeneity. As such it provides an explanatory framework to understand the origin of the lag-phase. In particular, we show that for any environmental condition there is a lag-phase that optimises fitness—the optimal duration of the lag-phase is negatively correlated to the amount of secondary nutrient in the environment. Using simulated evolution experiments we show that in stochastic environments evolved lag-phases depend on the distribution of nutrients over time[Table t1].

## Results

### Model

**Figure 1:**
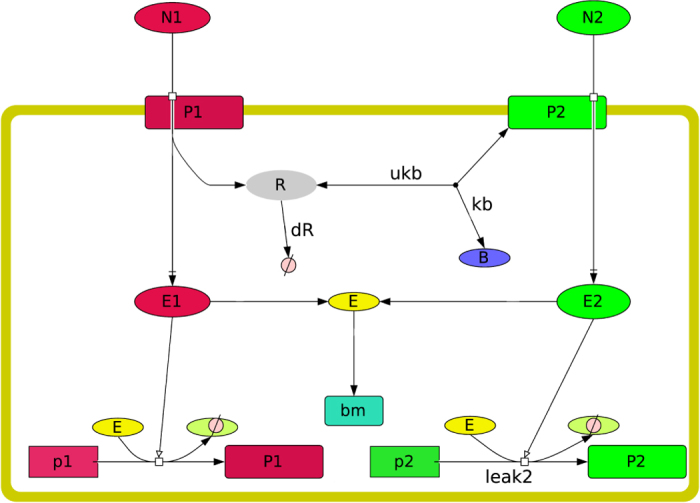
Schematic outline of the network model of a cell. The metabolic proteins *M*_1_ and *M*_2_ are omitted to increase readability. The key parameters, kb, ukb, dR are indicated in the diagram. The model equations are given in Table 2.

Our simulations are based on a stochastic biochemical network model of diauxic growth formulated in terms of chemical reactions. It represents gene regulation and expression, nutrient uptake and substrate degradation as well as cell growth and division (see Fig. 1 for a graphical representation and Tables 1 and 2 for an exhaustive list of the chemical reactions). To simulate the model we use an adaptation of the Gillespie algorithm^27^ extended to allow for growth and cell division (see Methods for details). The simulation software can be obtained from the authors on request.

The biochemical network of each cell allows for uptake of two external nutrients, *N*_1_ and *N*_2_. Throughout this article we assume that *N*_1_ is the primary (preferred) nutrient and can be thought of as glucose, whereas *N*_2_ represents a secondary nutrient, e.g. lactose. Uptake is mediated by the specific permeases *P*_1_ and *P*_2_. The external nutrient can only be taken up if the corresponding permease is present. Once inside the cell we denote the carbohydrate-phosphates by *E*_1_ and *E*_2_, i.e. *E*_1_ would represent Glc-6-P, or *N*_1_ that has been taken up. For the purpose of this article, we are not interested in details of the downstream metabolism, which we thus abbreviate by two reactions that convert *E*_1_ and *E*_2_ into some internal energy *E*. Crucial to our purpose here is that the primary nutrient is “better” than the secondary nutrient, i.e. it supports higher levels of growth. To reflect this in the model, we stipulate that *E*_1_, the primary nutrient, converts into twice as much energy *E* as the secondary nutrient.

Internal energy is used by the cell in two ways. Each molecule of *E* is either converted into “biomass” or it is used for the synthesis of the permeases (*P*_1_ and *P*_1_ and the metabolic enzymes *M*_1_ and *M*_2_). Biomass is best thought of as the energy surplus that the cell can generate from its nutrient uptake. The cell can divide once it has generated a sufficient amount of biomass (see below). At the same time, the cell also needs to invest into the uptake machinery and maintain sufficient levels of permeases and metabolic enzymes, or else it would not be able to process the nutrients sufficiently fast. This creates a resource allocation problem which will be the origin of the sensing cost for the cell.

The topology of our biochemical network model contains a mechanism for inducer exclusion similar to the PTS system. The crucial protein in the PTS system is dephospho-EIIA^Glc^ which represses the ability of *lac* permeases to take up lactose. In the model we implement this interaction in an abbreviated form. Uptake of the primary carbon source *N*_1_ coincides with production of *R* via de-phosphorylation. This repressor *R* takes the role of dephospho-EIIA^Glc^ in the PTS system during glucose transport and serves as an intra-cellular indicator of glucose uptake in close analogy to dephospho-EIIA^Glc^. Once produced, *R* is then either re-phosphorylated with a rate constant dR (reaction R.14 in Table 2) or it interacts with *P*_2_ to form the inactive molecule *B* (reaction R.15) with a rate constant kb; the reverse reaction happens with a rate constant ukb (reaction R.16). The formation of *B* models the interaction of dephospho-EIIA^Glc^ with the permeases of the secondary nutrients in real cells. Importantly, *B* does not take up nutrient, but is inert. Depending on the choice of parameters this reaction network can lead to a more or less efficient repression of the secondary uptake system in the presence of *N*_1_. When *N*_1_ is depleted, no more *R* is produced and the secondary metabolism becomes unrepressed.

Diauxic growth is often thought to depend on carbon catabolite repression (CCR) but the importance of CCR for the control of sequential uptake has become somewhat unclear^6^. In preliminary simulations we have established that direct repression of the secondary metabolism does not add any noticeably effects. For reasons of model parsimony, we have therefore decided to omit carbon catabolite repression from the model.

So far we described the properties of a single network representing an individual cell. Each cell can divide into a daughter and a mother cell when it has reached a certain threshold biomass. Any resource is then distributed randomly to mother and daughter cell. Thus a single founder cell can give rise to an entire population. As long as there is nutrient in the environment, an initial population of cells will tend to grow. The detailed rules for division and growth are described in the Method section.

### Parametrisation

Accurate parametrisation of computational models in biology, especially kinetic models, is often problematic. Numerical estimates of kinetic parameters are rare. When they are available, they are usually affected by large errors. In the present case this problem is avoided because we consider a model for a generic mechanism and not a species-specific network. We will not be trying to fit our model to a specific experiment, but instead we will be interested in generic, qualitative behaviours. As will become clear below, this means that we only need to consider relationships between parameter values, not absolute numbers. All parameter values to be used in this contribution will have been obtained from simulated evolution experiments, i.e. our algorithms were free to choose parameters within a given range. For practical reasons, we constrained the range for all parameters to the arbitrarily chosen, but fixed, interval [0, 15]. Only the rates of signalling interactions are multiplied by 100 (see reaction R.14–R.16 in Table 2). This reflects the fact that these reactions are much faster than gene expression reactions. As will become clear below, this does not imply that all signalling reactions are 100 times faster than the other reactions, it merely means that they can be chosen from a larger interval. Overall, the choice of the interval has the effect of setting an arbitrary (but fixed) time unit to the model. All parameters reported here have to be understood to be given in terms of this time-unit.

Feasible choices of external nutrient numbers are limited by considerations of computational cost. Since populations are growing exponentially, the corresponding demands on run-time and memory also grow exponentially. In our simulations we considered therefore up to 400000 nutrients only.

### Sequential uptake and the lag-phase are controlled by few parameters only

#### Sequential uptake is controlled by binding/unbinding ratio, dR and the leak expression

The model we are using here has 19 parameters that control expression rates and set time-scales. The quantitative behaviours of the model may be sensitive to the details of the parametrisation, including the choice of the random seed. In contrast, we found that the qualitative behaviour is robust, in the sense that (*i*) it is not difficult to find parameters that lead to decent growth of the population and (*ii*) small changes to these parameters tend to leave the qualitative behaviour unchanged. Regarding the central questions of this article, we found that most parameters of the model are uninformative. There is a large number of choices of parameters that are “functioning” in the sense that (*i*) they enable the population to exhaust both nutrients and (*ii*) lead to substantial (although not necessarily maximal) growth. For the present purpose we can consider these functioning parameter sets as equivalent, even though they vary in terms of their efficiency and achievable growth.

We found that the sequential nutrient uptake and the lag-phase (the phenomena of interest here) are controlled by a well defined sub-set of parameters only. Given any functioning set of parameters, when exposed to a dual nutrient environment the model can be tuned to simultaneous or strictly sequential uptake or anything in-between by adjusting a small number of parameters only. For a particular parameter set, this is illustrated in Fig. 3a.

Before we explain in detail how the lag-phase and sequential uptake are controlled by parameters, we first need to introduce a quantitative measure of the duration of the lag-phase. Visual inspection of growth curves alone is not sufficient to determine whether or not a population takes up nutrient sequentially. We therefore define the *delay measure*, a normalised (and dimensionless) indicator calculated as the ratio between the time to exhaustion of *N*_1_ and induction of *N*_2_ (see Methods for details and formula). If the delay measure is positive, then we consider uptake to be sequential, whereas a negative delay measure indicates a certain degree of simultaneous uptake. The maximal value of the delay measure is +1; the minimal value is −1. Higher values indicate a stricter sequential regime and long lag-phases.

Sequential uptake of nutrient relies on the repression of the *N*_2_ uptake machinery when there is *N*_1_ in the environment. The mechanism for such a repression is built into the reaction model via the reversible formation of *B* from the permease *P*_2_ and the repressor *R* (reaction R.15). Repression is only effective for some parameter values. We found that given any functioning parameter set, the lag-phase can be adjusted by tuning just a small number of parameters. These are: the phosphorylation rate dR, the *unbinding/binding ratio* (ratio of the unbinding rate and the binding rate of repressor and permease (ukb/kb)), and constitutive (base) expression of the permeases for the secondary metabolism, leak2.

The phosphorylation rate dR acts like a decay rate constant for *R*. It must be set sufficiently low so that there are enough repressor *R* molecules in the cell to bind to most of the secondary permeases *P*_2_. As long as this is the case then sequential uptake can be achieved by setting the unbinding/binding ratio low. If on the other hand the unbinding/binding ratio is high, then nutrient uptake will be simultaneous. Finally, leak2 will turn out to be an important determinant for the length of the lag-phase. At the same time, the leak expression is also linked to energy usage in that a higher leak expression implies an increased metabolic burden on the cell.

**Figure 2:**
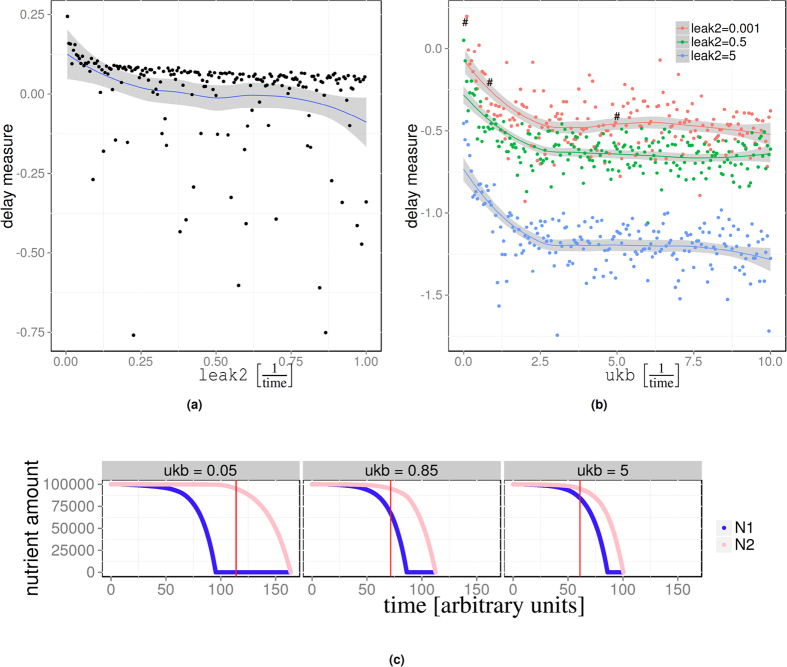
The influence of parameters on model behaviour. (**a**) The delay measure as a function of the leak expression of the secondary nutrient; each point corresponds to a simulation using a fixed parameters set with ukb = 0, kb = 15; see Supplementary Information for the remaining parameters. (**b**) The same parameters as in (**a**) but now we vary ukb for different choices of leak2. Increasing the unbinding rate of the repressor quickly leads to a vanishing delay. (**c**) Remaining carbon source over time for 3 different values of ukb. The red vertical bar indicates the point where *N*_2_ reaches 5% consumption. The three simulations correspond to the points labelled by hashes in (**b**).

We now describe in more detail the effect of these key-parameters on the outcome of the simulations. Example simulations illustrating the effect of the parameters are provided in Fig. 2. If the unbinding ratio is low then the association of *P*_2_ and *R* is fast compared to dissociation, the repression of the secondary metabolism during *N*_1_ uptake is efficient, and nutrient uptake is sequential. Once the primary nutrient is depleted no more repressor is produced and any remaining molecules of *R* are phosphorylated. At this point the secondary metabolism becomes de-repressed. This scheme works for all functioning parameter sets (provided dR is sufficiently low).

The efficiency of the repression is usurped by a large leak expression. Higher leak expression rates increase the probability of leaky import of *N*_2_ before the primary carbon source is depleted. In those situations the repressor *R* may not be able to bind all *P*_2_ that have been synthesised and repression becomes inefficient. Hence, high leak rates lead to higher leak-uptake of the secondary carbon source and therefore to a less efficient repression of the secondary nutrient at all unbinding/binding ratios. This is illustrated by the example simulations shown in Fig. 2b. For the particular parameters used there, positive delays could not be reproduced at all for the highest leak rate (leak2 = 5). For the lower leak rates a positive delay only emerges when ukb is low.

#### The lag-phase is controlled by the leak expression rate

**Figure 3:**
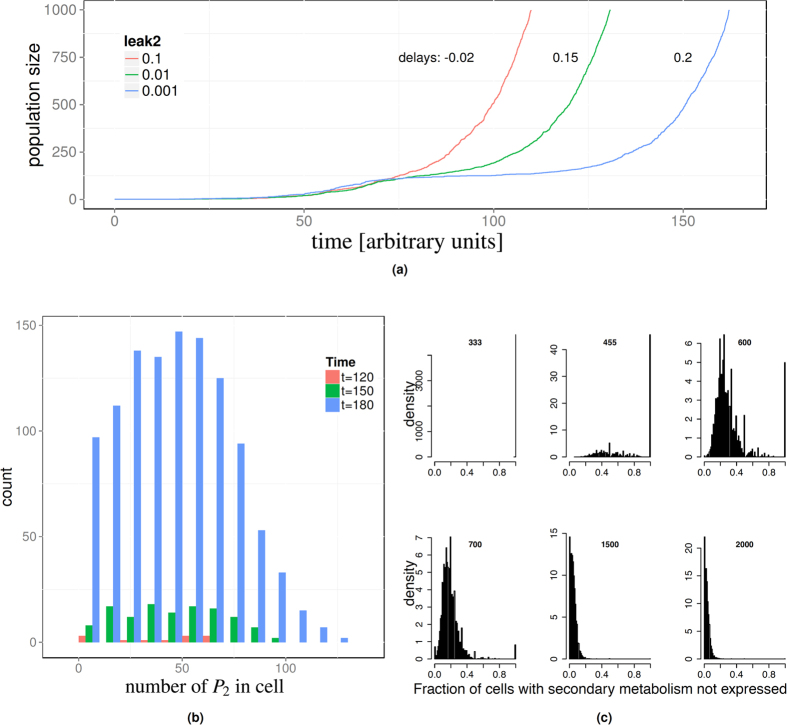
The lag-phase depends on the leak rate. (**a**) Three example simulations illustrating the lag-phase for leak2 = 0.1, 0.01, 0.001; the remaining parameters were fixed; see Supplementary Information for parameter values used. Here we used *N*_1_ = 80000 and *N*_2_ = 600000. (**b**) Distribution of the number of permeases in the population during the lag-phase for the simulation with the smallest leak rate in (**a**). We consider times *t* = 120, 150, 180 and only cells with at least one permease. Over time there is an increasing number of cells with more than one *P*_2_ permease. (**c**) Fraction of cells without a single *P*_2_ in a population after 100 time units. Each histogram represents 10000 simulation with a given saturation level of the permeases K; see main text for details. At low saturation levels (K = 333) the secondary metabolism is switched off and none of the cells expresses *P*_2_. Increasing the saturation point leads to bistable behaviour where the secondary metabolism is switched on sometimes only and then to all cells expressing *P*_2_.

Sequential nutrient uptake is a necessary but not sufficient condition for a lag-phase to emerge. When *N*_1_ and *N*_2_ are taken up sequentially, then the duration of the lag-phase is controlled by the leak expression of the secondary nutrient (Fig. 3a), more specifically the parameter leak2, which determines the constitutive background expression of *P*_2_.

To understand the effect of the leak rate on the duration of the lag-phase it is instructive to consider a scenario where there is no leak expression. Setting leak2 to zero will prevent the cells from inducing the secondary metabolism even if there is a substantial amount of secondary nutrient *N*_2_ in the environment. In this case lag-times would be infinitely long. Very high leak rates imply constitutive high level expression of the secondary metabolism, and hence no lag-phase. For small but non-zero leak rates the secondary metabolism will be switched on with a delay causing a lag-phase depending on the leak-rate.

The relationship between the leak rate and the lag-phase is highlighted by Fig. 3a which shows three different growth curves, all with the same parameters, but different leak rates. The highest leak rate leads to no visually discernible lag-phase while a significant lag-phase is apparent from the simulation with the lowest leak rate.

### During the lag-phase the population is heterogeneous

A consequence of the stochastic nature of the model is that individual cells may not switch to the secondary nutrient at all, even if all the primary nutrient is exhausted. Real world micro-organisms display a similar behaviour^18^. Within the model the failure to switch can be traced to the fact that the production of *P*_2_ requires internal energy *E*. At low leak rates there can be a substantial delay between the primary nutrient *N*_1_ being depleted and the activation of the secondary metabolism. In this regime the simulated cells continue to express the primary metabolic machinery. This consumes energy, but is futile because there is no more *N*_1_ to take up. During this period, the cell does not gain any nutrient. Consequently, cells can run out of internal energy *E*, leaving them unable to produce *P*_2_ and hence unable to switch. For those cells that do switch eventually (not all do), the average waiting time for the secondary metabolism to be induced is related to 1/leak2. This stochastic waiting time impacts the observable population-level growth (because only those cells that have already switched are able to grow) and manifests itself as a lag-phase.

When this is the case, then the growth during the diauxic shift is driven by those cells that successfully manage to switch to the secondary nutrient. Depending on the parameter settings, especially the leak rate, this may be only a small fraction of the overall population. All of the cells created after the diauxic shift are then descendants of this group while the other cells remain in a dormant state. A direct consequence of this is that the growth rate at the level of the population is reduced because only a sub-set of all cells grow. If one considers only population-level measurements of the growth rate (e.g. optical density over time) this manifests itself as a lag-phase and appears to indicate that cells have uniformly reduced their growth rate. In reality, it may be that the majority of the cells has ceased growth altogether, while a minority continues to divide at a maximum rate, which would have the same aggregate effect as a globally reduced growth rate. This is the case in the particular example simulations (shown in Fig. 3b) where only four cells had at least one permease for the secondary nutrient when the primary nutrient was exhausted. At higher leak rates more cells are able to switch to the secondary nutrient and the lag-phase is less pronounced.

In summary, in the model there are two effects that lead to a reduction of the population growth when the primary nutrient is exhausted. (*i*) A low leak rate means that cells need to wait longer for the secondary permease to be expressed. Once the secondary permease is expressed there is a delay before the relevant genes can be fully expressed. (*ii*) When the leak rate is sufficiently small, then a proportion of the population will not be able to switch at all and enters a dormant state. The overall growth rate will then be lower, manifesting itself as a lag-phase.

### Preparation is a natural consequence of stochastic activation

Wang *et al.*^17^ found that populations “prepare” for switching, i.e. anticipate the impending exhaustion of the primary nutrient and switch to the secondary nutrient before the primary nutrient is depleted. The same effect can be observed in our simulations.

Preparation could be achieved in two ways in the model. One mechanism is to reduce the efficiency of the repression of the secondary nutrient. This would result in a constant small uptake of *N*_2_. In this case, preparation is then simply the “leakiness” of the repression mechanism. In the model this is controlled by the unbinding/binding ratio. The higher the ratio the higher the leakiness (Fig. 2c).

Another, more interesting, way to achieve preparation is to repress the secondary metabolism efficiently until primary nutrient levels have reached a certain low threshold level. Only then is the secondary metabolism de-repressed. This mechanism of preparation requires the cell to “sense” external levels of primary nutrient. Once the number of *N*_1_ falls below the saturation threshold of the permease the uptake rate will reduce as external levels of nutrient deplete. In this regime the uptake rate is related to the concentration of the primary nutrient. The number of *R* (or EIIA^Glc^ in real cells) acts as a readout for measurements of this concentration. Reducing the amount of *R* in the cell will then lead to a weakening of the repression and, in due course, activate the secondary pathway.

In deterministic systems, it would be possible for this mechanism to de-repress the secondary metabolism suddenly at a given threshold concentration of *N*_1_. Real cells and our simulated cells are not deterministic, but are subject to random fluctuations. These fluctuations entail an effective uncertainty about the amount of repressor *R* in the cell and hence whether or not the threshold is crossed^28^. This uncertainty is a manifestation of well known limitations of stochastic sensors^29^.

Following from this sensing uncertainty, we would expect that for low concentrations of the primary nutrient parts of the population start to switch to the secondary nutrient, whereas parts of the population continue to grow exclusively on the primary nutrient. The result would be bi-stability with respect to the activation state of the secondary metabolism.

This second type of preparation can be reproduced in our model. The parameter controlling it is the saturation level of the permease for the primary nutrient, i.e. the parameter K. To demonstrate this, we modified the model so that the external nutrient is kept at a constant level. Then we set *N*_1_ and *N*_2_ to 100 and 100000 respectively and ran the model for 100 time units. For the final population, we then recorded the fraction of cells that had no *P*_2_ at all. We repeated this 10000 times for different saturation values of the permeases, i.e. different values of the parameter K (see R.3 and R.4 in Table 2). For the lowest value we considered (K = 333) not a single cell expressed *P*_2_. Similarly, for the highest value, (K = 2000) all cells express *P*_2_. In between we found that some of the cells had the secondary metabolism activated, whereas others had no *P*_2_ at all; this indicates a heterogeneous population and bi-stability (Fig. 3c).

### The optimal lag-phase depends on the frequency of environmental conditions

The lag-phase entails a substantial fitness penalty due to reduced growth during switching. However, a short lag-phase causes costs as well. Above, we have established that in our model the lag-phase is controlled by the leak expression of the secondary system. The higher the leak rate the shorter the lag-phase. Leak expression requires cell-resources of at least two kinds: (*i*) Protein synthesis draws metabolic resources (i.e. ATP, ribosome sequestration, etc.); (*ii*) inserting permeases at the cell surface requires space which is then not available to other cell functions.

This suggests that there is an optimal level of leak expression balancing the costs and benefits of short lag-phases. The location of this optimal lag-phase depends on the environment. In environments that contain only the primary nutrient there is no need for a secondary metabolism. In this case the optimal leak rate is zero, implying an infinite lag-phase. The opposite extreme is an environment that contains the secondary carbon source *N*_2_ only. In this case the secondary metabolism can be expressed constitutively and the primary metabolism never needs to be activated. For in-between cases the optimal duration of the lag-phase will increase with the amount of secondary nutrient *N*_2_ in the environment.

**Figure 4:**
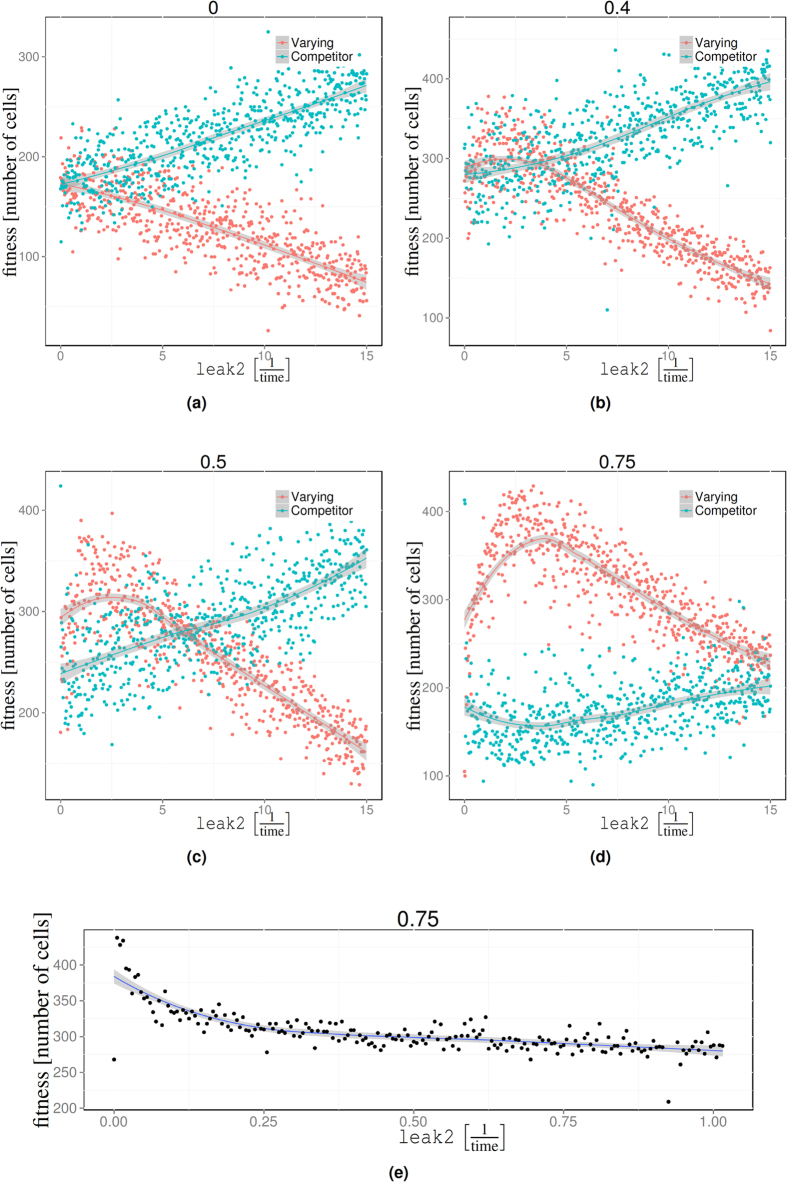
Fitness as a function of the leak expression of the secondary metabolism. (**a**–**d**). Each simulation was seeded with one cell of “Varying” and one cell with parameters “Competitor”. The two parameters were identical, except for the value of leak2 which varied along the horizontal axis. The parameters of “Competitor” remained constant throughout and are given in the Supplementary Information. Each point represents the number of cells at the end of a single growth batch. In all four graphs the same overall amount of overall energy is available, but distributed in a different way across *N*_1_ and *N*_2_. *N*_1_ was set to 200000, 120000, 100000, 50000 corresponding to the fractions of energy contained in *N*_2_/(*N*_2_ + 2*N*_2_) = 0, 0.4, 0.5, 0.75. The three parameters have optimal leak expressions located at ≈2.08, 2.46, 3.98 respectively. The competitor is negatively affected by successful varying solutions. (**e**) The fitness as a function of the leak expression rate in the absence of competition and 75% of the energy contained in *N*_2_. There is no optimal leak expression.

To confirm this expectation we performed competitive simulations in mixed environments. We seeded the model with two parameter sets. Both sets were identical to begin with. However, for one of the parameter sets (labelled “Varying”) we varied the leak rate (parameters leak2 and mleak2) from 0 to 15. The other parameter set (labelled “Competitor”) we kept fixed throughout. This latter parameter set is in direct competition for nutrients with the “Varying” set. Using this set-up we then performed a number of simulations where we varied the leak expression rate (Fig. 4) of the “Varying” set. Then we recorded the number of cells belonging to the two sub-populations after all nutrients had been consumed. We did this for four different nutrient compositions (Fig. 4b–d). These simulations showed that there is a leak rate that optimises competitiveness. The position of the optimum depends on the relative amount of the primary and secondary nutrient. Since the leak-rate determines the length of the lag-phase, this result entails that there is an optimal lag-phase depending on the particular mixture of nutrients in the environments.

Note that the existence of an optimal lag-phase crucially depends on the presence of a competitor. When a lineage does not compete for nutrients with some other “species,” then the optimal leak rate is zero with fitness decreasing with increasing leak (Fig. 4a). In this single-lineage case, there is no benefit for the cells to switch rapidly because the available nutrient is not consumed by anybody else, yet the cost of switching rapidly remains. Hence, it is best to minimise the leak expression as much as possible (Fig. 4e).

### Evolution *in silico*

To understand better how the parameter settings and fitness are related, we extended our simulation model to include evolution of parameter sets. We limited the evolutionary process in our model and only allowed the parameters but not the topology of the network itself to evolve. To achieve this we implemented an evolutionary scenario where parameter sets (“cells”) compete with one another for nutrients and are subject to random mutation events (see Methods for details). During the evolution, we allowed all parameters to vary. The only exception was the capacity of the surface to accommodate permeases (parameter KL; c.f. R.5–R.8 in Table 2). This parameter determines the number of permeases that corresponds to half of the capacity of the surface. It sets how much space there is for permeases on the cell surface and is therefore not an evolvable trait, but depends on the physical dimensions of the cell.

Here we report evolutionary simulations under two different environmental regimes. Firstly, we performed simulations with mixed nutrient environments, that is environments always contained the primary and secondary nutrient albeit in varying proportions (see Methods for details of the simulation protocol). We evolved solutions for 4 different surface capacity parameters, corresponding to the values KL = 5, 20, 50, 100. For each value of KL we performed 4 independent evolutionary runs, i.e. altogether we performed 16 simulations of evolution. From each of the evolutionary runs, we obtained a population of different parameters sets adapted to the respective conditions (see Methods for details on how we chose the solutions).

**Figure 5:**
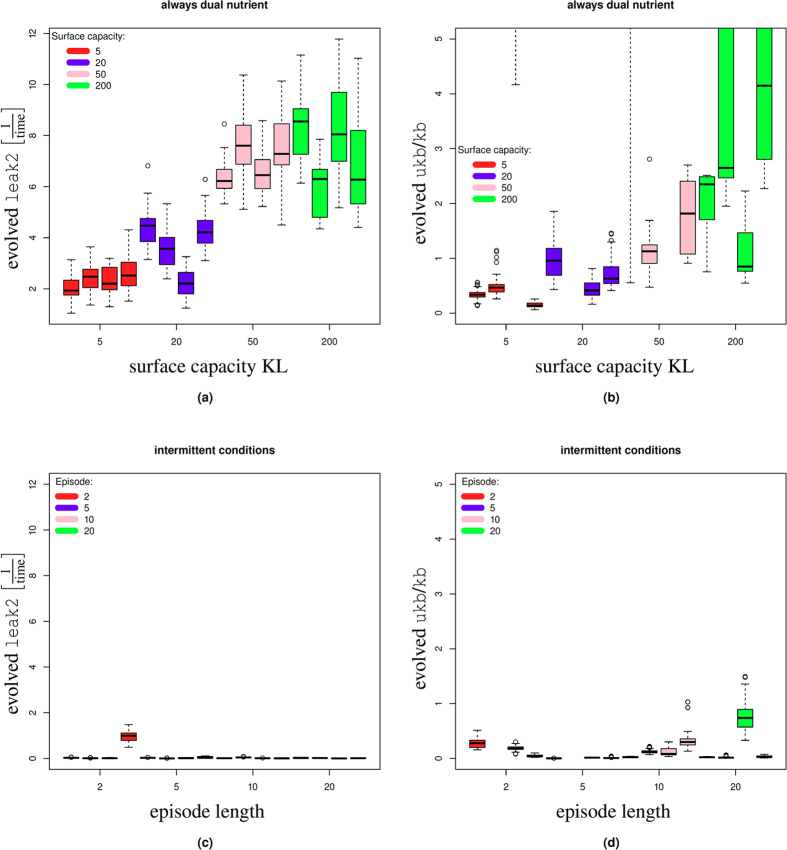
The evolved values for leak expression and the unbinding ratio. (**a**) Environments always carried dual nutrients. The boxplot shows the parameters obtained from 4 evolutions for 4 different surface capacities. Lower capacities tend to favour lower leak rates. (**b**) The same simulations as in (**a**), but we show the unbinding ratio. (**c**,**d**) Here we show parameters that evolved under mono-nutrient conditions that were occasionally intermitted by dual nutrient conditions. The longer the episode length the rarer the dual nutrient conditions appear.

For all KL considered here we found that the evolved leak rates and unbinding ratios were considerably larger than zero; this means that in none of the evolutionary experiments we observed a strong pressure towards sequential uptake. Furthermore, the evolved parameter values were also distributed over a wide range (Fig. 5a,b).

The parameter KL has a strong impact on the evolved leak rate leak2 (see Fig. 5b). The difference between the two larger values KL = 50 and KL = 200 is not significant; yet for KL = 20 and KL = 5 the evolved leak rates are much lower compared to simulations with higher capacities. The correlation between KL and the unbinding/binding ratio is less pronounced (see Fig. 5b). For each of the three lowest surface capacities considered, there was at least one evolutionary run where the evolved average unbinding ratio was >5 (and outside the graph); hence sequential uptake did not evolve in all cases.

Altogether, we found that the adaptive pressure for sequential uptake depends on the capacity of the cell to metabolise nutrients as determined by the parameter KL. Small capacities tend to favour the evolution of parameters where the overlap between uptake of primary and secondary nutrient is less pronounced, i.e. they exert adaptive pressure to longer delays (Fig. 5b). This relationship between the surface capacity KL and sequential uptake is to be expected based on the following consideration: When the metabolic capacity of the cell is unlimited then simultaneous uptake of the secondary nutrient would not reduce the ability of the cell to grow on the primary nutrient. There would then be no adaptive pressure for sequential nutrient uptake. As the surface capacity reduces the relative impact of a given amount of leak uptake becomes larger. In the case of very small uptake rates, even a small amount of leak expression of the secondary nutrient leads to a large percentage drop of the uptake rate of *N*_1_ and a corresponding drop in the growth rate. In this regime it is beneficial to repress the secondary metabolism efficiently because the relative impact of leak expression on the growth rate is higher when the overall capacity is low.

Secondly, we considered an *intermittent* environment. In this environment the cells were confronted with episodes of mono-nutrient conditions intermitted by dual-nutrient conditions (see Methods for details). The effect of this pattern is that often the cells have no need to switch between nutrients, because there is only one nutrient in the environment. We use the episode length *L* to control how often during the evolution a new population is grown in a dual nutrient environments. The higher *L* the rarer dual-nutrient environments occur; see Methods for details. We considered four different episode lengths *L* = 2, 5, 10, 20 (Fig. 5). For all these choices, all simulations evolved leak2 values close to zero and low unbinding ratios (Fig. 5c,d). Both indicative of a strong sequential use of nutrients and long lag-phases. However, for the two lowest episode lengths, there were two outlier unbinding/binding ratios with values >5 (and not visible on the graph). This indicates that for low ratios the evolution of inducer exclusion becomes unstable and there is an increased probability that it will not emerge.

**Figure 6:**
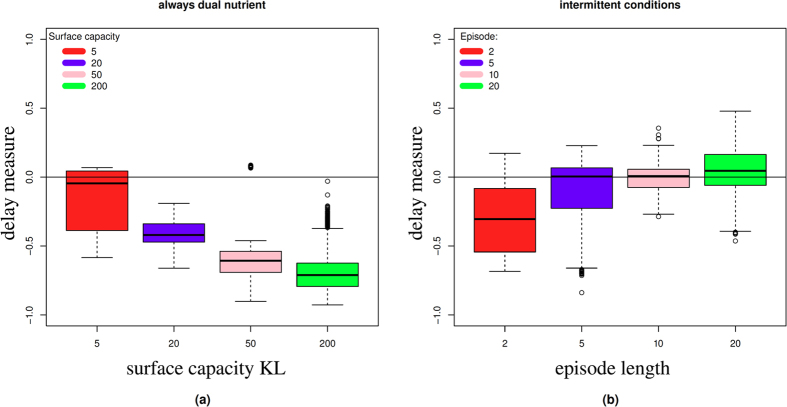
(**a**) Actual delay measure for the solutions evolved under dual nutrient conditions corresponding to Fig. 5a,b. For each evolved parameter set we performed 100 simulations to determine the delays. Each boxplot shows actual delay values obtained from the most numerous genotype of the simulated evolution experiments and represents 400 simulation results. (**b**) Delay measure for parameters evolved under intermittent conditions corresponding to Fig. 5c,d.

In order to understand the actual behaviour of the evolved solutions, we took the most common parameter set from each of the four evolutionary runs. Then we simulated these parameter sets 100 times each (with the initial conditions *N*_1_ = *N*_2_ = 100000 and no evolution). For each of those simulations we then calculated the delay measure to understand the evolved lag-phase. Figure 6 shows the boxplots of the resulting delays corresponding to the evolution experiments from Fig. 5.

There are stochastic variations between runs of the same parameters and hence, for each parameter set, there is a distribution of behaviours, not a single result (Fig. 6). In the mixed environment delay measures remain predominantly negative indicating a tendency for concurrent uptake and no lag. The higher the capacity the lower the delay measure, i.e. the greater the overlap between *N*_1_ and *N*_2_ uptake. Positive delay measures are obtained only for the smallest capacity, although the mean value is still negative. We only obtain positive average delay measures for the case of the intermittently mixed simulations and for longer episode lengths (Fig. 5c). In the case of the intermittently mixed simulations we find that delay measures increase with episode length, but there are large variations between simulations with the same parameters.

Altogether, it emerges that there is a relationship between the statistical properties of the environment and the strength of the sequential uptake that evolves: For environments that consistently present both the primary and secondary nutrient, the delay between primary and secondary nutrient is minimal. On the other hand, when there are long stretches of single nutrient conditions, only occasionally intermitted by dual nutrient environments, then a stronger repression of the secondary nutrient evolves. This result is also in qualitative agreement with previously reported wet-lab evolution experiments^8^.

## Discussion

Sequential nutrient uptake is a manifestation of resource limitations of the biological cell and can be predicted based on metabolic flux optimisation^30^,^31^ or simple considerations of competitive dynamics^13^ and is therefore unproblematic evolutionarily. The evolutionary origin of the lag-phase is less clear. While the phenomenon itself can be understood as a consequence of population heterogeneity during switching^18^, the evolutionary driver of the lag-phase is not known. Here we proposed an explanatory *ansatz* for the lag-phase as a direct consequence of the maintenance cost of the sensing apparatus for external nutrient. This provides a novel and parsimonious evolutionary rationale for the lag-phase during diauxic growth that is ultimately rooted in the stochastic dynamics of the cell and well known biophysical limitations of sensing^25^,^26^.

For our simulations we reduced the metabolic pathways to a few reactions and included only elementary regulation to mimic the inducer exclusion mechanism. Yet, these few interactions are sufficient to predict the empirically known trade-off between the length of the lag-phase and the long-term growth of the cell. This trade-off arises as a consequence of the cost of biological sensing, pointing to a cost-efficiency trade-off of biological sensors as the ultimate cause of the lag-phase.

Our conclusions are likely not limited to species that fit the particular reaction scheme that we assumed here. The trade-off between cost and accuracy of sensors is valid in general^25^,^26^, independent of any assumptions about the underlying biochemical network, and hence so are the conclusions we reach. At the same time, since our model is generic, it does not allow us to make quantitative predictions about any specific species. Consequently, we cannot conclude that cost-accuracy trade-offs of sensors are the only or indeed the dominant driver of the evolution of the lag-phase. It is likely that there are other drivers. Ultimately, this is an empirical question and it is to be expected that the importance of the sensor costs for the lag-phase are species dependent.

Conceptually, the link between sensing and the duration of the lag is the preparation phase. Ideally, the cell switches to the secondary nutrient immediately when all *N*_1_ is depleted, but not before, i.e. ideally there is no preparation phase. Given this ideal scenario the cells would be able to maximise uptake of *N*_1_. This would presuppose that it is possible to detect immediately and accurately when the primary nutrient is exhausted. In the stochastic cell sensing always comes at a cost to the cell. Various schemes of passive sensing^32^ which solely rely on the free energy contribution from outside are no exception. In those case the accuracy of the sensor is still limited by the number of receptors^26^. Receptors in turn need to be maintained which entails a resource cost on the cell.

In the particular case we consider here the sensing problem is reduced to a binary result, i.e. deciding whether or not the external nutrient is present. The relevant accuracy measure is then the sampling frequency of the sensory system, i.e. the leak expression rate of the secondary permease which determines for how long the cell needs to wait before it has sufficient permease to induce the secondary metabolism.

The simulations presented above demonstrate that a higher leak rate reduces the lag-phase, but increases the length of the preparation phase and thus causes a premature activation of the secondary metabolism. This is sub-optimal because it leaves high quality nutrient for competitors to consume. Additionally, a leak rate being a constitutive background expression causes costs to the cell at all times and therefore reduces achievable growth rates irrespective of the carbon source. Hence, there is a trade-off between fast switching and fast growth.

This poses the following dilemma for the cell: It can minimise the impact of the dramatic, but episodic, lost growth during the switch from one carbon source to another. If it does so, then it necessarily decreases its long-term growth prospects. Empirically, this antagonistic tension between the ability to grow rapidly and to adjust to environmental conditions is well known in the experimental literature^18^,^22^. Here we showed that one would expect this based on biophysical considerations of sensing cost only.

Our model represents two types of cost: (*i*) The sensing apparatus (i.e. permeases) requires space on the surface and thus occupies valuable “real estate.” (*ii*) Proteins need to be constantly re-synthesised to avoid degradation through dilution, entailing a direct metabolic cost and a resource drain on the cell machinery for example via sequestered ribosomes^33^. In the particular set-up and parametrisation of our model, the former is the main contributor (see Fig. 4a) while synthesis costs of leak expression are small.

The balance between spatial and metabolic cost in real cells is an empirical question and must be assumed to be species dependent^34^. We conjecture that spatial costs can be substantial. *E. coli* , for example, has specialised permeases for about twenty different carbon sources. In addition there are permeases for amino acids and other molecular types that need to be embedded into the cell surface. The minimal number of permeases required to induce the *lac* operon is 300^35^ corresponding to approximately 0.4% of the cell surface. This is not significant by itself. However, in order to guarantee that a given proportion of the cells has this minimal number, the average coverage needs to be much higher. The same reasoning applies to any of the other permeases. Competition for space between the various uptake systems is therefore likely to limit leak expression rates.

Taking all the elements together, the following overall picture emerges: Consistent with previous empirical findings^8^,^19^ our model showed that there is an optimal lag-phase for a particular mix of nutrients in the environment. Realistic environments are rarely constant but typically present complex sequences of conditions over time. For each of those successive conditions there is an optimal leak rate. Hence, the cell must maximise fitness over the distribution of conditions that form its environment. In realistic environments the best lag-phase is the one that balances, for a given distribution of environmental conditions, long-term growth with the ability to switch rapidly as the environment changes. We then arrive at an interpretation of the lag-phase as an adaptation to the statistical properties of a particular environment. Stable environments will tend to favour strains that grow fast. In environments where the nutrient composition changes frequently, it will be more important to avoid long delays due to lag-phases.

In this context it is interesting to note that some investigators found apparent bet-hedging strategies, whereby a population had a mixture of lag-phases^20^. Rather than adapting to the average environment, a bet-hedging strategy would equip sub-populations to respond differently to changing environments. Such strategies have been shown to be evolutionarily stable^36^. We did not observe bet-hedging, as such, to evolve in our model. A likely reason for this is that the population sizes we can simulate are too small. However, as noted above, for certain conditions that are favourable for a strong repression of the secondary metabolism, this repression did not reliably evolve. In particular this is evident from Fig. 5d; for some conditions both very low and very high unbinding ratios evolved. This could indicate the beginning of a bet-hedging strategy.

## Methods

### Delay measure

Our model is stochastic and therefore it is not possible to prevent a certain level of leak-uptake of *N*_2_ even for very efficient repression of the secondary metabolism. Assuming equal initial amounts of both nutrients, we consider the secondary metabolism as inactive when 5 or less percent of *N*_2_ are taken up; we consider uptake to be sequential when the *N*_2_ metabolism is inactive by the time *N*_1_ is depleted. This 5% threshold value is arbitrary and chosen mainly to facilitate discussion of the results. Based on this threshold, we define the *delay measure* as the time between *N*_1_ being exhausted and the *N*_2_ metabolism being activated, divided by the time since simulation start to activate the secondary metabolism. All delays reported here were determined with the initial condition *N*_1_ = *N*_2_ = 100000. If this delay measure is negative, then we take this to mean that the secondary metabolism was activated before the first nutrient was used up. On the other hand, if this time is positive then we interpret this as sequential uptake.

### Simulations

The model is simulated as a set of *N* × *m* chemical reactions where *N* is the number of cells in the system and *m* the number of reactions per cell. The user specifies the *m* reactions characterising the cell model (Table 2) and these are instantiated *N* times with the parameter sets of the individual cells. An implementation of Gibson and Bruck’s version of the stochastic simulation algorithm^38^ was used to manage the reaction system as reaction propensities vary.

Division happens when a cell has reached a threshold biomass. Biomass is produced from internal energy *E* (reaction R.1) and is itself modelled as a discrete molecular entity not a continuous trait. The division threshold size is set to 50. Division is modelled as a pseudo-reaction event and proceeds with a rate of 1. Upon division, a new daughter cell is created and *m* new reactions are added to the reaction system. All molecules and resources of the dividing cell, including permeases and metabolic proteins, nutrients and energy, and “biomass” are randomly (but not necessarily equally) split between mother and daughter cell.

Cell division may leave either mother or daughter in a deadlock state where they have no permeases left nor any resource to produce permease. This could be, for example, if one of the cells receives no internal energy *E* and hence remains unable to start its processes. Deadlock states may also happen during normal non-division processes through stochastic fluctuations. In such cases, the cells are, in effect, dead and removed from the simulation. Note that cells that have no *E* but contain a number of permeases are considered dormant and will not be removed.

Permeases *P*_*i*_ and metabolic proteins *M*_*i*_ are not available in constant numbers but need to be expressed by the cell from their genes. Gene expression is modelled as a one-step process, as the precise dynamics of transcription/translation is not relevant for the present purpose. Protein synthesis consumes internal energy *E* and can only proceed when *E* is available (see reactions R.7–R.10). The regulation in the model is based on a common bacterial de-repression motif. The permeases *P*_1_, *P*_2_ and metabolic proteins *M*_1_, *M*_2_ are permanently repressed by a repressor protein which is displaced by their respective internalised nutrient *E*_1_ and *E*_2_. The internal repressor is not explicitly modelled and de-repression is represented as activation assuming Hill kinetics. Throughout we assume a Hill coefficient of 2 for the Hill kinetics. The reasoning for this choice is as follows. A Hill coefficient of 1 (or smaller) does not support the bi-stability between the primary and secondary nutrient^13^,^39^. Hill coefficients above 4 become quickly unrealistic biologically. There is little qualitative difference between low Hill coefficients. Hence, it is reasonable to fix the Hill coefficient at 2 throughout (see Supplementary Information).

The cell can express both *P*_1_ and *P*_2_ with leak rates leak1 and mleak1 respectively for the metabolism of the primary nutrient; analogously the leak rates for the secondary metabolism are leak2 and mleak2 (see reactions R.5–10). This leak rate represents constitutive expression in the absence of a de-repressor. Regulation of genes and uptake of nutrient is modelled by Hill functions. Again we assume a Hill exponent of 2 throughout.

A cell has a user-definable limit on the capacity for permeases on its surface. This capacity is formulated as a Hill repressor function with the Hill constant KL representing half capacity. This repressor function gives the probability that a permease that has been produced will not be integrated into the surface. In the model this is implemented through futile reactions consuming nutrient, but producing no permease (R.5 and R.6).

### Evolution

**Figure 7:**
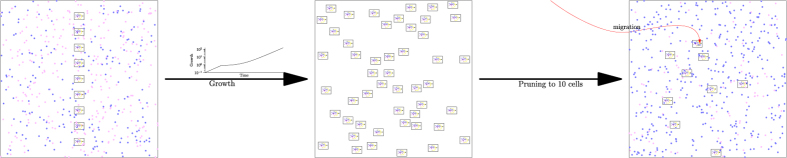
Artificial evolution. A simulation is initialised with 10 random solutions. This seed population is then allowed to grow. Once all nutrient is used up it is pruned to 10 seed cells and another growth batch is started.

The evolutionary runs were performed by starting the model with a seed population of 10 cells with randomly assigned parameters drawn from a uniform probability distribution with interval [0, 15]. All subsequent cells were grown (Fig. 7) from these initial 10 cells using the standard model. The only difference was that during evolutionary runs daughter cells were occasionally subject to a random *mutation* event; that is, one of its parameter values was changed by a small amount, potentially affecting the fitness of the cell. The environmental conditions consisted of either only *N*_1_ or a random mix of *N*_1_ and *N*_2_. In all cases the composition of the nutrient always contained the same amount of usable energy, i.e. could be converted into the same number of *E*. A growth batch lasted until all of the nutrient supplied at the beginning had been used up. At this point 10 cells were randomly selected to form the founder population of a new growth batch; this is the step that implements selection. Note that the cells in each of the seed population are not necessarily different from one another and may contain several copies of the same parameter set.

Altogether, 5000 such growth batches were completed for each artificial evolutionary simulation. In order to achieve a larger final population and a more efficient evolutionary dynamics, we ran 32 environments/populations in parallel. Each of these 32 parallel environments were independent from one another except for occasional migration events between them. Migration only took place during the seeding phase, where cells were moved to a random new environment. Each cell in the 32 populations was chosen to migrate with a probability *p* = 0.001; i.e. one in one thousand cells was selected for the seed population of a different random environment.

#### Intermittent and mixed nutrient environments

We considered two different types of environments for our evolutionary simulations. (*i*) A mixed environment that always contained the primary and the secondary nutrient. After each growth batch the nutrient was replenished with a different mix of primary and secondary nutrient while keeping the total energy in the environment constant between subsequent growth batches; the initial nutrient compositions was identical across parallel environments. (*ii*) An environment with episodes of *L* growth conditions containing only *N*_1_. After each episode, a fair coin was tossed to decide whether the following episode consisted of another *L* batches with *N*_1_ only, or a single batch with mixed nutrients. After that we tossed another coin and so on. A typical sequence of environmental conditions for *L* = 2 could be: 11M11M1111M11M …, where “ 1” represents an environment consisting of *N*_1_ only and “ M” represents a mixed environment. We tested four different episode lengths with *L* = 2, 5, 10, 20.

We performed the evolution for 5000 batches. We found by initial tests that a higher number of batches did not lead to materially different results. At the end of these 5000 batches we then obtained a population of parameters sets from across the 32 parallel environments. Within this large final population we then selected all the individual parameter sets that had at least 50 copies. This latter condition removed all the fresh and untested mutants that had arisen during late stages of growth. We considered this reduced set as the final population.

### Software

The simulation software used is developed in house using Java. It is based on the Gillespie algorithm, but augmented to encapsulate internal states of cells as well as cell division and death. The system as a whole is represented as a large system of biochemical reactions. Variations of cell numbers are achieved by dynamically adding and removing reactions during the run-time of the simulation, as described above.

The software can simulate a single population without evolution or full evolutionary runs with arbitrary numbers of populations. In addition to the implicit evolution algorithm, the software also allows the use of genetic algorithms to evolve parameters sets that optimise a user-defined fitness function (although this feature has not been used for the present contribution). Extended evolutionary runs are computationally expensive, but the software supports parallelisation of populations on multi-core machines or cluster computers running Sun Grid Engine.

The Java source code of the main simulator and additional scripts can be obtained from the authors on request.

## Additional Information

**How to cite this article**: Chu, D. and Barnes, D. J. The lag-phase during diauxic growth is a trade-off between fast adaptation and high growth rate. *Sci. Rep.* **6**, 25191; doi: 10.1038/srep25191 (2016).

## Supplementary Material

Supplementary Information

Supplementary Dataset

## Figures and Tables

**Table 1 t1:** Explanation of the main variable names and parameters used in the model.

(a)	
Symbol	Description
*N*_1_	external primary nutrient, e.g. glucose
*N*_2_	external secondary nutrient, e.g. lactose
*E*_1_	internal primary nutrient, e.g. Glc-6-P
*E*_2_	internal secondary nutrient, e.g. allolactose
*E*	internal energy
*P*_1_	permease specific to *N*_1_
*P*_2_	permease specific to *N*_2_
*R*	repressor of primary uptake
**(b)**	
**Param**	**Description**
leak2	leak rate of permease expression
ukb	dissociation rate of *B*
kb	association rate repressor, permease
dR	phosphorylation rate of repressor
KL	capacity of cell surface for permeases
K	half capacity of permeases
K_X_	half saturation of gene activation for *P*_x_
g	rate of converting *E* into biomass

**Table 2:** System of reactions for a single cell.

Substrate	Product	Rate	
5 *dol*	biomass	20	R1
*E*	*dol*	g	R2
*N*_1_	*E*_1_ + *R*	*P*_1_ hill(*N*_1_, K, 2)	R3
*N*_2_	*E*_2_	*P*_2_ hill(*N*_2_, K, 2)	R4
*E*		(leak1 + a1 hill(*E*_1_, K1, 2)) (hill(*P*_1_ + *P*_2_, KL, 2))	R5
*E*		(leak2 + a2 hill(*E*_2_, K2, 2)) (hill(*P*_1_ + *P*_2_, KL, 2))	R6
*E*	*P*_1_	(leak1 + a1 hill(*E*_1_, K1, 2)) (1-hill(*P*_1_ + *P*_2_, KL, 2))	R7
*E*	*P*_2_	(leak2 + a2 hill(*E*_2_, K2, 2)) (1-hill(*P*_1_ + *P*_2_, KL, 2))	R8
*E*_1_ + *E*	*E*_1_ + *M*_1_	(mleak1 + ma1 hill(*E*_1_, mK1, 2))	R9
*E*_2_ + *E*	*E*_2_ + *M*_2_	(mleak2 + ma2 hill(*E*_2_, mK2, 2))	R10
*M*_1_ + *E*_1_	*M*_1_ + *E*	*M*_1_ hill(*E*_1_, lk1, 2)	R11
*M*_2_ + *E*_2_	*M*_2_ + *E*	*M*_2_ hill(*E*_2_, lk2, 2)	R12
*M*_2_ + *E*_2_	*M*_2_	*M*_2_ hill(*E*_2_, lk2, 2)	R13
*R*		100 · dR · *R*	R14
*R* + *P*_2_	*B*	100 · kb · *R* · *P*_2_	R15
*B*	*R* + *P*_2_	100 · ukb · *B*	R16

We abbreviate the expression *x*^*z*^/(*x*^*z*^ + *y*^*z*^) byok hill (x, y, z). All parameters are limited to the interval [0, 15], but note the factor of 100 in the last three reactions; see main text for details.
