# Supplementary material for: The lag-phase during diauxic growth is a trade-off between fast adaptation and high growth rate
Source: Sci Rep. 2016 Apr 29;6:25191. doi: 10.1038/srep25191 (PMC4850433; doi:10.1038/srep25191)
Supplement: Supplementary Information [file srep25191-s1.pdf]

The lag-phase during diauxic growth is a trade-off between fast adaptation and high growth rate
Supplementary information

Dominique Chu and David J. Barnes

Importance of Hill exponent

Here we investigate check the impact of the Hill coefficient on the general behaviour of the model. We performed two types of simulations. In all simulations in the main article we assumed that the Hill exponent has a value of 2. We chose this as the minimum Hill exponent that supports bistability in deterministic systems.

For the first set of simulations we use the parameters from the “Competitor” in fig. 5a. We varied the Hill exponent between 1.0 and 4.0 in steps of 0.1 performing 2000 simulations for each for each value. Each simulation was started with a single cell with the initial conditions $N_1 = N_2 = 100000$ and no evolution. The population size was measured when both types of nutrient were exhausted. Figure 1 shows the resulting fitness for various exponents. There is no noticeable difference between the values.

In a different set of simulations we tested random parameter sets for four different Hill exponents. Figure 2 shows the distribution of fitnesses for random parameters for the Hill exponents 1,2,3 and 4 based on 10000 simulations for each exponent. The four resulting distributions are not significantly different. Hence, we conclude that the choice of Hill exponent is immaterial for our purpose, for as long as it supports bistability, i.e. is larger or equal 2.

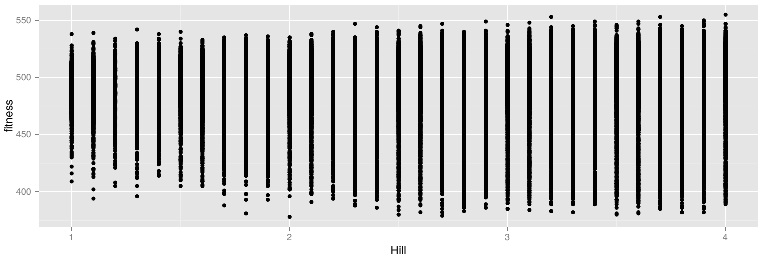

Figure 1: The distribution of fitness for different Hill exponents using a fixed parameter set.

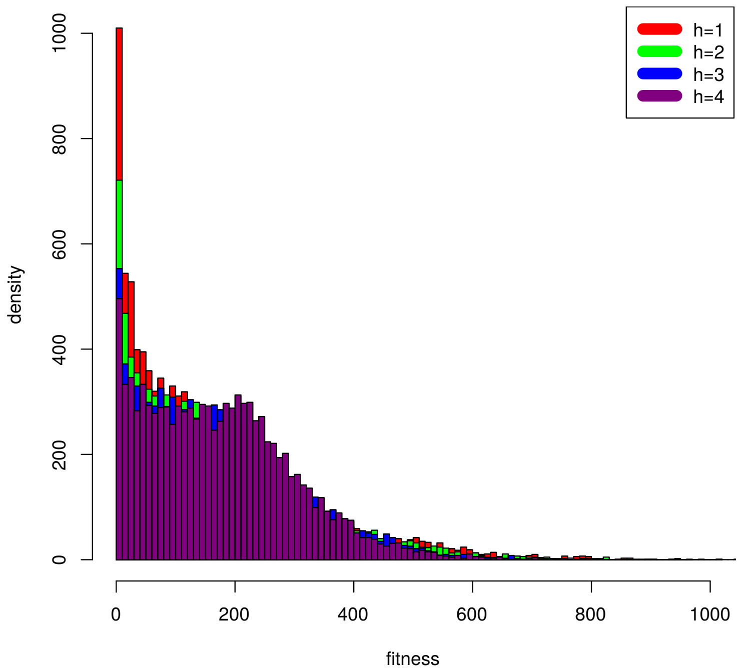

Figure 2: The distribution of fitness for random parameters and different Hill exponents.
